# Vision Development Differences between Slow and Fast Motor Development in Typical Developing Toddlers: A Cross-Sectional Study

**DOI:** 10.3390/ijerph17103597

**Published:** 2020-05-20

**Authors:** Elena Pinero-Pinto, Verónica Pérez-Cabezas, Concepción De-Hita-Cantalejo, Carmen Ruiz-Molinero, Estanislao Gutiérrez-Sánchez, José-Jesús Jiménez-Rejano, José-María Sánchez-González, María Carmen Sánchez-González

**Affiliations:** 1 Department of Physiotherapy, University of Seville, 41009 Seville, Spain; epinero@us.es (E.P.-P.); jjjimenez@us.es (J.-J.J.-R.); 2 INDESS (Instituto Universitario para el Desarrollo Social Sostenible), Department of Nursing and Physiotherapy, University of Cadiz, 11009 Cadiz, Spain; carmen.ruizmolinero@uca.es; 3 Department of Physics of Condensed Matter, Optics Area, University of Seville, 41012 Seville, Spain; mhita@us.es (C.D.-H.-C.); jsanchez80@us.es (J.-M.S.-G.); msanchez77@us.es (M.C.S.-G.); 4 Department of Surgery, Ophthalmology Area, University of Seville, 41009 Seville, Spain; egutierrez1@us.es

**Keywords:** child development, motor skills, vision disorders, evaluation, physical therapy, optometry

## Abstract

Many studies have established a relationship between visual function and motor development in toddlers. This is the first report to study two-year-olds via an assessment of their visual and motor skills. The purpose of this study is to describe the possible changes that can occur between visual and motor systems in typical developing toddlers. A total of 116 toddlers were included in this observational, descriptive, and cross-sectional study. Their mean age was 29.57 ± 3.45 months. Motor development variables studied were dominant hand/foot; stationary, locomotion, object manipulation, grasping, visual motor integration percentiles; gross motor, fine motor, and total motor percentiles; and gross motor, fine motor, and total motor quotients. Visual development variables were assessed including visual acuity, refractive error, ocular alignment, motor fusion and suppression, ocular motility, and stereopsis. Our findings demonstrated that typical developing toddlers with slow gross motor development had higher exophoria and further near point of convergence values compared to toddlers with fast gross motor development (*p* < 0.05). No statistically significant differences were found in visual acuity and stereopsis between slow and fast gross motor development toddlers.

## 1. Introduction

Visual and motor development involves a physical and physiological process that allows perceiving precise details of an image (eyesight) and a perceptual process (vision) that requires multisensory integration: vision, hearing, touch, and proprioception for the interpretation of visual information. Vision delivers a key sensory input required for the proper functioning of neural circuits and is therefore a brain process of sensory integration [1] that offers important information in most daily activities [2]. Binocular vision offers improvements over monocular vision. It allows precise depth perception (stereopsis). It also enables the exploration of the visual scene during movement planning of the hands and feet directed at a target in space, such as the hand to grasp items [3] and the feet to walk [4], climb obstacles [5], or even for sitting postural control [6]. Three- and six-month-old babies can better assess if objects are within reach and move toward them using both eyes instead of one [7], as demonstrated during crawling [8].

Toddlers follow a pattern of skill development that allows them to know when they are progressing adequately. The first years of life are critical to a child’s overall development. The central nervous system grows at a very fast rate, similar to the skills that the child develops. During this period, the development of postural reflexes and reactions, basic motor skills, and gross voluntary motor skills lay the foundation for mature motor behavior [9]. Many developmental tests are based on motor activity, such as the Peabody Developmental Motor Scale-II (PDMS) [10]. Gross motor development assesses muscle control, coordination, and locomotion. Fine motor skills include the development of control and coordination of body segments to perform more precise and complex tasks, integrating muscle coordination and perceptual skills [11]. Motor development can be influenced by other areas of development above and beyond sensory systems during childhood [12] For example, babies learns motor skills through observation [13].

Many studies have established a relationship between visual function and motor development [14,15,16,17,18,19]. They claim that binocular vision disorders such as amblyopia and strabismus can adversely affect skills that depend on eye movements, including reading. Many of these studies assessed only fine motor skills or eye-hand coordination in school-age children and their relationship to amblyopia. They studied groups of participants with different age ranges, between 5–9 years [14], 8–12 years [15,16,17], and 10–30 years [18]. Other research determined that children with poor stereoacuity have significant visuomotor deficits compared to typical developing children [20], while other reports affirmed that stereoacuity may be limited to specific tasks. In a group of children aged 5 to 13 years without visual impairment, Alramis et al. [21] showed that binocular vision and stereoacuity were associated with higher performance of certain fine motor tasks, and task performance decreased in younger children. They concluded that the role of vision in the performance of fine motor skills depends on both the task and age.

In addition to fine motor skills, few studies measured postural stability and control or gross motor skills in typical developing children. Some reported a delay in motor development and skill acquisition in children with visual disabilities and described a delay in gross motor skills such as control of the head and ability to sit, crawl, and walk during the first year of life [12,22]. Souza et al. [23] observed that a group of 15- to 22-month-old toddlers with visual impairment presented an overall delay in neuropsychomotor development, mainly in coordination. Celano et al. [24] suggested that there may have been a delay in fine motor function and balance in a group of 4.5-year-old children with unilateral visual impairment secondary to congenital cataracts. Chakraborty et al. [25] demonstrated a close relationship between stereopsis and fine and gross motor skills in a group of 4.5-year-old toddlers. Thompson et al. [26] studied a large group of 2-year-olds to fully evaluate the state of vision (visual acuity, stereopsis, alignment of visual axes, eye motility, and self-refraction) and its possible relationship with motor development. Their results demonstrated that global perception of movement and binocular vision are associated with motor function at an early stage of development as measured by the Bayley Scale of Infant and Toddler Development 3rd edition (BSID-III). All of the scientific literature reviewed included babies and toddlers [12,22,23,24] with some form of visual impairment or born with risk factors for neurological development [25,26]. To the best of our knowledge, this is the first report to study visual and motor development in a sample of between two and three-year-olds both visually and from the perspective of motor development.

The purpose of this study was to describe the vision development differences between slow and fast motor development in typical developing toddlers. Vision study included ocular health screening, visual acuity, refractive errors, ocular alignment, motor fusion and suppression, ocular motility and stereopsis. Among motor development parameters, we studied fine motor development through grasping and hand-eye coordination and gross motor development, analyzing static, locomotion, and object manipulation.

## 2. Materials and Methods

### 2.1. Design

This observational, descriptive, and cross-sectional study was conducted from October 2019 to January 2020 in toddlers at the facilities of the University of Seville’s (Spain) nursery schools. This study followed the Declaration of Helsinki’s tenets. The parents were written and orally informed about the study characteristics, benefits, and risks. Written informed consent was obtained after explaining the nature and possible consequences of the study. The Institutional Review Board of the University Hospital Virgen Macarena of the University of Seville approved this research.

### 2.2. Subjects

The sample consisted of 116 typical developing participants. Inclusion criteria were toddlers with normal or corrected-to-normal vision, and exclusion criteria were toddlers with previous history of disorder, toddlers with learning disabilities, and preterm toddlers.

### 2.3. Measurements and Materials

A physiotherapist’s measurements were blinded to an optometrist’s measurements and vice versa. A first examination was conducted in an individual room. After one hour, second measurements were completed. If the toddlers reported signs of fatigue, they were given a 30-min break before the second set of measurements was obtained.

### 2.4. Developmental Motor Assessment

The Peabody Developmental Motor Scale-Second Version (PDMS-II), the motor subscale [10] was used to evaluate motor development. This scale evaluates gross and fine motor development in toddlers from birth to five years. The gross motor component included three subtests for toddlers ages two to three: stationary (standing balance, sit-ups, and push-ups), locomotion (walking, running, jumping, and hopping), and object manipulation (throwing and taking different size balls). The fine motor component is composed of two subtests, grasping and visual motor integration. The mean between both scales is considered the total motor development score. The test requires the child to perform specific motor activities that are scored with a 2, 1, or 0 depending on whether the child partially or correctly completes an activity according to the description. Results obtained are standard scores, percentiles, age equivalents, quotient scores in fine and gross motor areas, and total motor development.

The mean value of the development quotients is 100 [10]. Above 100 (quotient > 100), motor development is considered rapid and below 100 (quotient < 100), development is considered slower. We presented two groups, toddlers with fast gross motor development (quotient > 100) and a group of toddlers with slow gross motor development (quotient < 100) based on the variable gross motor quotient (GMQ). The entire PDMS-II can be finished in 45 to 60 min. Separate fine or gross motor subtest administration takes 20 to 30 min.

The PDMS-II is primarily designed to examine and evaluate motor development, but as a secondary objective, it was developed as a research tool. The general test administration procedure is standardized, and formal training is not required. Griffiths et al. [27] reviewed the characteristics and psychometric properties of different tools for evaluating motor development. They determined that the PDMS-II has good psychometric characteristics to evaluate motor development. Hua et al. [28] reported that the scale’s internal consistency is excellent. Folio and Fewwell [10] determined that it is good (24–35 months, α = 0.97). Wuang et al. [29] reported that the test has good reliability (test-retest *n* = 141, ICC = 0.97), and excellent validity. The content and the structural validity are also excellent [10,28]. The minimal detectable change was 7.76 (sensitivity 60.65%, specificity 74.13%) [29] and standard error mean (SEM) for 24–59 months was 3 [10].

### 2.5. Visual Development Assessment

#### 2.5.1. Visual Acuity and Refraction

External structures of the eye were examined with a pen flashlight and head loupe. Red reflex testing [30] was measured with a direct ophthalmoscope with the lens power set at 0. The red reflex brightness should be identical in both eyes. Any absence of the red reflex, difference between the eyes, or abnormal pupil color may indicate a serious eye condition. The quantification of visual acuity was measured using Cardiff cards [31] and broken wheel tests [32]. Refractive errors were assessed with the non-cycloplegic method and measured with Mohindra retinoscopy [33]. The retinoscope light was the only stimulus for the toddler and did not induce accommodation. The unexplored eye was occluded, and the optometrist was 50 cm from the child. Shadows observed were neutralized with positive or negative spherical or cylindrical lenses. The spherical equivalent (SE) was calculated to homogenize the refraction variables.

#### 2.5.2. Ocular Alignment

Very young toddlers, such as our toddlers, cannot fixate on a target long enough for a valid cover test. In such cases, the optometrist estimated the degree of ocular alignment with the corneal reflex test and diopter prisms (the Krimsky test) or without diopter prisms (the Hirschberg test and kappa angle) [34]. The kappa angle was described as the angle between the visual axis and pupil axis. Each eye has a different Kappa angle, usually less than five degrees. A positive Kappa angle (displacement toward the nose) is physiologic up to five degrees. A negative kappa angle represents a temporal displacement (toward the ear). A large kappa angle may cause ocular alignment disorders [35]. Angles that were zero or no differences between the visual and pupil axis were reported as centered.

#### 2.5.3. Motor Fusion, Suppression, and Stereopsis

For convergence/divergence test, a fixed target was presented at 25 cm. First, a 20∆ base-out (BO) prism was placed in front of one of the child’s eyes. Next, a 20Δ base-in (BI) prism was placed in front of one of the child’s eyes. The child should direct his/her hand in front of or behind the fixed target [36]. The near point of convergence (NPC) was determined by placing a fixed target 30 cm from the eye in the midplane of the child’s head. The child was asked to maintain fixation on the target. The toddlers were asked to describe the picture that they looked at during the measurement. The target was moved slowly toward the eyes until one eye lost fixation and turned out. The distance between the fixed target and the nose bridge was measured with a string and ruler. It was repeated twice for each child [37,38]. To test ocular motility using binocular fixation, the toddler fixed on a small dot for 20 s [17]. Next, smooth movement without restrictions was assessed via smooth pursuit to a moving target located 30 to 40 cm away [36]. Saccades eye movements were also studied. The toddlers were instructed to look at a target point as quickly and accurately as possible using their index finger. Target appeared randomly at four eccentricities ± 5 degrees or ± 10 degrees from central fixation in the horizontal plane [39]. The Lang stereo test II was used to measure stereopsis. The test consisted of three three-dimensional images, a moon, truck, and elephant, and one two-dimensional image, a star that is seen without stereoscopic vision (visible with only one eye) that serves to capture the patient’s attention. The test was placed with the observer in front of the child to observe his/her eye movements. The toddlers were told to look at the picture lying perpendicular to approximately 40 cm from the child’s face and asked if he/she saw anything, observing the eye movements. If the child was unable to name the images, he/she was asked to locate an area on the card where there appeared to be something different and try to describe their differences [40].

### 2.6. Statistical Analysis

The data were analyzed with SPSS statistical software (version 26.0 for Windows; SPSS Inc., Chicago, IL, USA). Descriptive analysis was conducted with values expressed as mean ± SD. The data normality distribution was assessed with the Kolmogorov-Smirnov test. Gross, fine, and total motor quotients were divided into two groups (fast motor development when the GMQ, FMQ, and TMQ was ≥ 100 and slow motor development when the GMQ, FMQ, and TMQ was 100). Differences in visual development between the fast and slow motor development groups were assessed with Student’s t-test for quantitative variables and the chi-squared test for qualitative variables. For all of the tests, the level of significance was established at 95% (P < 0.05).

## 3. Results

**Figure 1:**
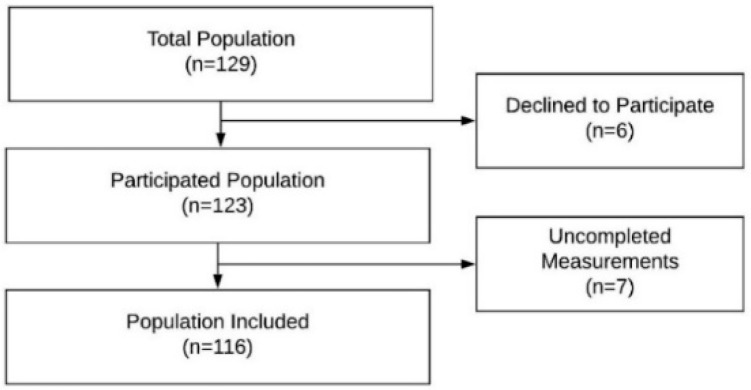
Population flow chart diagram.

This study included 116 toddlers, 53 males (45.68%) and 63 females (54.31%). The mean ages of the toddlers were 29.57 ± 3.45 (24.16 to 36.90) months. Population flow chart diagram is shown in Figure 1. In the gender comparison, no statistically significant differences were found in any variable except for the fine motor quotient (FMQ) where the males toddlers obtained 100.56 ± 16.64 and the females toddlers obtained 108.09 ± 12.21 (t = 2.79, *p* < 0.01). Motor development variables included the dominant hand and foot, stationary percentile, locomotion percentile, object manipulation percentile, grasping percentile, visual motor integration percentile, gross motor percentile (GMP), fine motor percentile (FMP), total motor percentile (TMP), gross motor quotient (GMQ), fine motor quotient (FMQ), and total motor quotient (TMQ). They are presented in Table 1. Visual development variables included the Cardiff visual acuity (VA) test for right, left, and both eyes; broken wheels VA test for right eye, left eye, and both eyes; retinoscopy refraction in the mean spherical equivalent; kappa angle for right and left eyes; Hirschberg reflex for right and left eyes; near point of convergence; base-out and base-in test; Lang stereopsis test; Bruckner test; fixation, accuracy, and head tests for tracking movements; and reflection and head tests for saccades movements. These are also presented in Table 1.

Visual development differences between the fast and slow GMQ development groups (GMQ > 100 and GMQ ≤100, respectively) are presented in Table 2. The GMP demonstrated identical results for the GMQ groups. We found no significant differences in visual development between the fast and slow FMP, TMP, FMQ, and TMQ development groups. In the right eye kappa angle, only 17.5% of the toddlers had a centered reflex in the slow gross motor development group (GMQ ≤ 100), while 36.8% of the toddlers had a centered reflex in the fast motor development group (GMQ < 100) (χ² = 8.28, P = 0.01). Similar results were found in the right eye Hirschberg test: only 17.95% of the toddlers had a centered corneal reflex in the slow motor development group, whereas 39.5% of the toddlers had the same type of corneal reflex in the fast motor development group (χ² = 7.64, P = 0.02). The same situation was found in the kappa angle and Hirschberg test for the left eye, although the results were not statistically significant (χ² = 4.74, P = 0.09, χ² = 4.88, and P = 0.08, respectively).

**Figure 2:**
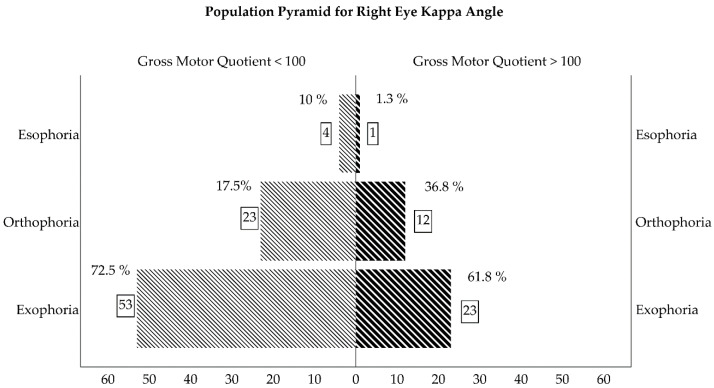
Population pyramid for kappa angle (expressed in percentage and count of toddlers in square).

**Figure 3:**
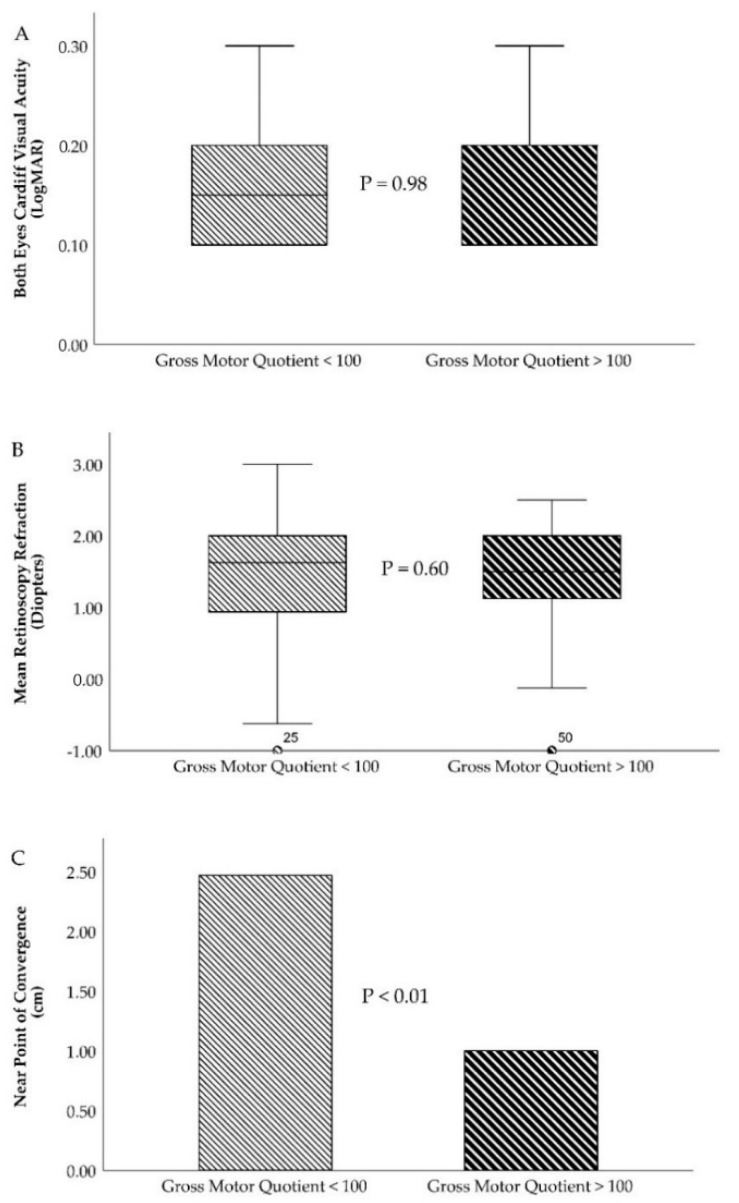
Slow and fast motor development characteristics for main optometry variables. (**A**)—Box and plot graph for both eyes’ visual acuity (Cardiff test, expressed in LogMAR). (**B**)—Box and plot graph for mean retinoscopy refraction (expressed in diopters). (**C**)—Near point of convergence (expressed in cm).

The Krimsky and Bruckner tests obtained similar findings. In the slow motor development group, 87.5% and 88.8% of the toddlers had a normal test while 12.5% and 11.3% had a deviated test, respectively. However, in the fast motor development group, all of the toddlers had normal Krimsky (Figure 2) and Bruckner tests and none had any deviations in their eyes (χ² = 4.92 and P = 0.02 for the Krimsky test and χ² = 4.39 and P = 0.03 for the Bruckner test). The last statistically significant visual development variable was near point of convergence (Figure 3C). The slow motor development group had 2.46 ± 4.07 cm, whereas better results were reported in the fast motor development group, 1.00 ± 2.02 (t =2.56, P = 0.01). For visual acuity (Figure 3A), retinoscopy refraction (Figure 3B), base-out and base-in test, stereopsis test, fixation test, accuracy and head position for tracking movements, and reflection and head position for saccades movements, there were non-statistically significant differences between slow and fast motor development including gross, fine, and total percentiles and quotients.

## 4. Discussion

This study evaluated the visual function and motor development in typical developing toddlers. The objective was to determine the possible presence of visual dysfunction and/or motor disorders and analyze possible differences between slow and fast motor development for visual system variables. Visual acuity, alignment of visual axes, stereopsis, and ocular motor skills were included in the present study. The percentiles and quotients of gross, fine, and total motor development in a group of 116 toddlers aged from two to three years were also reported. Two main GMQ groups were established, a slow motor development group (GMQ < 100) and fast motor development group (GMQ > 100). We found statistically significant differences between slow and fast motor development for certain visual system variables. Toddlers with slow gross motor development had a greater tendency of exophoria and a further near point of convergence (NPC) than toddlers with fast gross motor development. These findings agree with previous studies that demonstrated the link among the visual and motor systems in babies, toddlers, and children [4,13,14,15,16,17,19,21,24,25,26].

Similar to our outcomes, Thompson et al. [26] studied a two-year-old large toddlers group. They fully evaluated vision state (visual acuity, stereopsis, visual axes alignment, ocular motility, and auto-refraction) and its possible relationship with motor development. They measured using the Bayley Scale of Infant and Toddler Development 3rd edition (BSID-III), which has an excellent correlation with the PDMS-II [41,42]. Their outcomes revealed that global movement perception and binocular vision were associated with motor function at an early development stage. This study included toddlers born with risk factors for neurological development. Thus, their early development rates differ from ours since our sample was based on toddlers with neurotypical development. To the best of our knowledge, our study is the first that analyzed typical developing toddlers two- to three-year-old both in motor and visual function.

To date, the prevalence of visual, accommodative, and non-strabismic binocular dysfunction has increased in the pediatric population [43,44,45]. Early diagnosis and proper management can improve vision-related life in this population, thus guaranteeing its correct evolutionary development in all areas. Different symptoms and signs can be used to diagnose visual function [46,47].

### 4.1. Visual Acuity Differences between Slow and Fast Gross Motor Development

Amblyopia refers to the unilateral or bilateral reduction of the best corrected visual acuity that is not directly attributed to a structural alteration of the eye or visual pathways [48]. It constitutes one of the main causes of visual impairment in children, with prevalence values ranging from 1% to 4% [49]. Amblyopia is related to the presence of strabismus, refractive errors, astigmatism, and anisometropia. Determination of visual acuity is generally the first clinical step to identify the presence of amblyopia [50]. When we classified the subjects according to their GMQ values, we found no differences in visual acuity values between the two groups. Visual acuity was identical in the RE and LE (0.18 ± 0.10 LogMAR) measured with the Cardiff test, and there were no statistically significant differences between both eyes (OD = 0.36 ± 0.05 LogMAR, OI = 0.37 ± 0.04 LogMAR) when evaluated with broken wheels. Similar findings were obtained in binocular visual acuity values and did not differ between the two groups. Many studies link visual acuity deficits with motor delays [14,15,16,17,21,51,52]. The presence of refractive errors, mainly hyperopia, is common in children with amblyopia, which is normally associated with mild delays in many aspects of development [53]. In our study, the sample consisted of toddlers without visual impairment whose objective refraction did not exceed +1.50 diopters in either of the two eyes, which could be why we did not find differences between the slow and fast motor development groups.

### 4.2. Ocular Alignment Differences between Slow and Fast Gross Motor Development

Heterophoria is ocular misalignment in the absence of fusional vergence. Exophoria is a divergent misalignment, while esophoria is a convergent misalignment [54]. The measurement of heterophoria is an important clinical test since it indicates the demand of the fusional vergence system [55]. It has been fully evaluated in adults and older children, but is difficult to measure in toddlers younger than five years because of their limited cooperation [38,54,56,57]. We found authors who established that many toddlers under five years old were orthophoric [38,56]. However, our results are in line with more recent studies showing that the phoria of children without visual impairment between two and seven years old was mainly exophoric, with small differences in that age range [54,55,57].

Our study found that all of toddlers had clear exophoria, which significantly increased their slow motor development. Overall, 72.5% in the slow gross motor development group had a right eye kappa angle that was more positive (exophoric) than 61.8% in the fast development group (P = 0.01). The left eye kappa angle had significant differences (P = 0.09). Correspondingly, the right eye Hirschberg reflex was significantly more nasal (exophoric) (P = 0.02) and considerable (P = 0.08) in the slow motor development toddlers. Furthermore, the slower gross motor development toddlers had an NPC of 2.46 ± 4.07 cm, significantly higher than in the fast-gross motor development group, where the NPC was 1.00 ± 2.02 cm (P = 0.01).

The findings of Jeon et al. [58] were very revealing. They reported that the prevalence of exotropia was higher in toddlers with several motor impairments. Motor function was studied using gross motor function. In this case, our patients were typical developing, and we did not have cases of strabismus but had cases of exophoria. In this sense, the slow motor development group had greater exophoria than the fast motor development group. In addition, we found a decrease in the ability to merge with an NPC increase in the slow gross motor development group. These results agree with Jeon et al. [58]; however, they found the same prevalence in exotropia and esotropia.

On the one hand, exophoria is characterized by a divergent deviation in the line of sight when the visual axes are at rest. Exotropia is defined as a more serious, manifest, and fixed situation of ocular exodeviation [59]. On the other hand, Barbosa et al. [60,61] categorized infants by the degree of motor performance in three motor levels: cerebral palsy, motor delay, and typical development. Therefore, our results could be very useful for anticipating below average cases of motor development in which a higher prevalence of exophoria is demonstrated.

### 4.3. Stereopsis Differences between Slow and Fast Gross Motor Development

Stereopsis allocates depth calculations based on the binocular disparity between the images of an object in the left and right eyes [62]. Toddler stereopsis determination allows rapid detection of visual disturbances, mainly cases of amblyopia with a history of strabismus [63]. Previous studies concluded that toddlers with deficient stereopsis have developmental disorders [20]. Other studies showed that stereopsis is associated with higher performance of certain fine motor tasks at very young ages [21]. We also found studies that showed that many toddlers perform well on manual skill assessments even with poor stereopsis [64]. Previous research demonstrated that toddlers who underwent strabismus surgery showed postoperative improvements in motor performance that were not correlated with stereopsis improvements [65]. Our stereopsis findings were similar in the toddlers’ fine and gross motor development. This was probably because the toddlers in the sample did not present any type of visual alterations. Stereopsis was positive in both groups, with a slight non-statistically significant difference in the fast-gross motor development group (282.35 ± 131.35) second arc, compared to the slow gross motor development group (303.89 ± 143.67) second arc with P = 0.45.

### 4.4. Future Research and Limitations

Future research could be assessed by visual and therapy programs. Visual therapy has been shown to be a useful treatment option in subjects with visual disturbances [66,67] and assesses how it affects motor development quotients. We also proposed the reverse option, conducting an intervention to promote and enhance motor development in toddlers with slow gross motor development to ascertain how it affects binocular vision development. Within the limitations, sample follow-up was missing because this was a cross-sectional study. Future research should include longitudinal gross and fine motor development changes in this sample. The toddlers enrolled were in their last year of nursery school. This issue could limit sample follow-up.

## 5. Conclusions

Neurotypical developing toddlers and without visual impairment with slow gross motor development had higher exophoria and further near point of convergence values compared with fast gross motor development toddlers. No statistically significant differences were found in visual acuity and stereopsis between both slow and fast gross motor development toddlers.

## Figures and Tables

**Table 1:** Population characteristics for motor and visual development.

Motor Development Parameter (*n* = 116)	Value	Visual Development Parameter (*n* = 116)	Value		
			Right Eye	Left Eye	Both Eye
Age (Months)	29.57 ± 3.45 (24.16 to 36.90)	Visual Acuity (Cardiff Test—LogMAR)	0.18 ± 0.10 (0.10 to 0.70)	0.18 ± 0.10 (0.10 to 0.70)	0.17 ± 0.10 (0.10 to 0.70)
Dominant Hand (Right/Left)	113 (97.4%)/3 (2.6)	Visual Acuity (Broken Wheels—LogMAR)	0.36 ± 0.04 (0.20 to 0.40)	0.37 ± 0.49 (0.20 to 0.40)	0.37 ± 0.49 (0.30 to 0.40)
Dominant Foot (Right/Left)	109 (94%)/7 (6%)	Retinoscopy Refraction (Diopters D) Spherical Equivalent Refraction	+1.30 ± 0.85 (−1.00 to +3.00)	+1.39 ± 0.87 (−2.00 to +3.00)	-
Static Percentile	72.04 ± 19.90 (9.00 to 99.00)	Kappa Angle (Negative/0/Positive)	5 (4.3%) 35 (30.2%) 76 (65.5)	5 (5.2%) 34 (29.3) 76 (65.5)	-
Locomotion Percentile	15.87 ± 11.08 (2.00 to 50.00)	Hirshberg Reflex (*n* = 115 y 114) (Temporal/Centered/Nasal)	4 (3.4%) 37 (31.9%) 74 (63.8%)	5 (4.3%) 38 (32.8%) 71 (61.2%)	-
Handling Percentile	43.43 ± 21.20 (5.00 to 95.00)	Krismky Test (Normal/Deviated)	106 (91.4%)/10 (8.6%)		
Grasp Percentile	73.53 ± 24.16 (5.00 to 99.00)	Near Point of Convergence (centimeter, cm)	2.01 ± 3.62 (0.00 to 20.00)		
Coordination Percentile	37.79 ± 18.76 (2.00 to 84.00)	Base-Out 6∆ Prism Test (prism diopters, ∆) (Negative/Positive)	43 (37.1%)/73 (62.9%)		
Thick Motor Percentile (TMP)	42.40 ± 21.00 (8.00 to 95.00)	Base-In 6∆ Prism Test (prism, diopters, ∆ (Negative/Positive)	86 (74.1%)/30 (25.9%)		
Fine Motor Percentile (FMP)	56.68 ± 24.33 (12.00 to 99.00)	Stereopsis Lang Test (Second arc) (200”, 400” y 600”)	297.29 ± 139.77 (200.00 to 600.00)		
Overall Motor Percentile (OMP)	49.71 ± 22.32 (4.00 to 96.00)	Bruckner Test (Normal/Deviated)	107 (92.2%)/9 (7.8%)		
Thick Motor Quotient (TMQ)	96.81 ± 9.15 (79.00 to 124.00)	Fixation Test (Passed/Not passed)	90 (77.6%)/26 (22.4%)		
Fine Motor Quotient (FMQ)	104.52 ± 14.90 (14.00 to 151.00)	Accuracy and Head (Tracking Movements)	Smooth 34 (29.3%)/Loss 45 (38.8%)/Jumps 27 (23.3%)/Continuous Loss 10 (8.6%) Motionless 17 (14.7%)/Slight 49 (42.2%)/Medium 32 (27.6%)/Strong 18 (15.5%)		
Overall Motor Quotient (OMQ)	98.66 ± 14.55 (0.00 to 126.00)	Reflection and Head (Saccades Movements)	Negative 67 (57.8%)/Positive 49 (42.2%) Motionless 15 (12.9%)/Slight 54 (46.6%)/Medium 30 (25.9%)/Strong 17 (14.7%)		

Values were presented with mean ± SD (standard deviation) and (Range) in quantitative variables or expressed with frequency and percentage in qualitative variable.

**Table 2:** Visual development differences between over and under mean quotient for gross motor quotient development.

Visual Development Parameter	Gross Motor Quotient < 100 (*n* = 80)	Gross Motor Quotient > 100 (*n* = 36)	*p* Value *
Cardiff VA (RE)/(LE)	0.18 ± 0.10/0.18 ± 0.10	0.18 ± 0.10/0.18 ± 0.09	0.98/0.98
Broken Wheels VA (RE)/(LE)	0.36 ± 0.05/0.36 ± 0.04	0.37 ± 0.04/0.37 ± 0.04	0.26/0.34
Retinoscopy Rx (RE)/(LE)	+1.27 ± 0.91/+1.35 ± 0.92	+1.35 ± 0.73/+1.49 ± 0.74	0.66/0.43
Kappa Angle (RE) (Negative/0/Positive)	4 (10%) 7 (17.5%) 29 (72.5%)	1 (1.3%) 28 (36.8%) 47 (61.8%)	0.01
Kappa Angle (LE) (Negative/0/Positive)	4 (10%) 8 (20%) 28 (70%)	2 (2.26%) 26 (34.3%) 48 (63.2%)	0.09
Hirschberg Reflex (RE) (Temporal/Centered/Nasal)	3 (7.7%) 7 (17.95) 29 (74.4%)	1 (1.3%) 30 (39.5%) 45 (59.2%)	0.02
Hirschberg Reflex (LE) (Temporal/Centered/Nasal)	3 (7.9%) 8 (21.1%) 27 (71.1%)	2 (2.6%) 30 (39.5%) 44 (57.9%)	0.08
Krismky Test (Normal/Deviated)	70 (87.5%) 10 (12.5%)	36 (100%) 0 (0%)	0.02
NPC (centimeter, cm)	2.46 ± 4.07	1.00 ± 2.02	0.01
Base-Out 6∆ Prism Test (prism diopters, ∆) (Negative/Positive)	27 (33.8%) 53 (66.3%)	16 (44.4%) 20 (55.6%)	0.27
Base-In 6∆ Prism Test (prism, diopters, ∆ (Negative/Positive)	60 (75%) 20 (25%)	26 (72.2%) 10 (27.8%)	0.75
Stereopsis Lang Test (Second arc) (Negative, 550”, 600” and 1200”)	303.89 ± 143.67	282.35 ± 131.35	0.45
Bruckner Test (Normal/Deviated)	71 (88.8%) 9 (11.3%)	36 (100%) 0 (0%)	0.03
Fixation Test (Passed/Not passed)	64 (80%) 16 (20%)	26 (72.2%) 10 (27.8%)	0.35
Accuracy (Tracking Movements) (Smooth/Loss/Jumps/Continuous Loss)	22(27.5%)/33(41.3%) 20(25%)/5(6.3%)	12(33.3%)/12 (33.3%) 7(19.4%)/5(13.9%)	0.44
Head (Tracking Movements) (Motionless/Slight/Medium/Strong)	12(15%)/32(40%) 24(30%)/12 (15%)	5(13.9%)/17(47.2%) 8(22.2%)/6(16.7%)	0.82
Reflection (Saccades Movements) (Negative/Positive)	46 (57.5%) 34 (42.5%)	21 (58.3%) 15 (41.0%)	0.31
Head (Saccades Movements) (Motionless/Slight/Medium/Strong)	12(15%)/33(41.3%) 24(30%)/11(13.85)	3(8.3%)/21(58.3%) 6(16.7%)/6(16.7)	0.23

VA: Visual Acuity; NPC: Near point of convergence; RE: Right eye; LE: Left eye. Quantitative value with mean ± SD and qualitative with frequency (percentage).
